# Sars-CoV-2 Virus Infection May Interfere CD34+ Hematopoietic Stem Cells and Megakaryocyte–Erythroid Progenitors Differentiation Contributing to Platelet Defection towards Insurgence of Thrombocytopenia and Thrombophilia

**DOI:** 10.3390/microorganisms9081632

**Published:** 2021-07-30

**Authors:** Mario Giosuè Balzanelli, Pietro Distratis, Gianna Dipalma, Luigi Vimercati, Alessio Danilo Inchingolo, Rita Lazzaro, Sergey Khachatur Aityan, Maria Elena Maggiore, Antonio Mancini, Rita Laforgia, Angela Pezzolla, Diego Tomassone, Van Hung Pham, Donatello Iacobone, Annalisa Castrignano, Antonio Scarano, Felice Lorusso, Silvio Tafuri, Giovanni Migliore, Angelo Michele Inchingolo, Kieu Cao Diem Nguyen, Tran Cong Toai, Francesco Inchingolo, Ciro Gargiulo Isacco

**Affiliations:** 1 SET-118, Department of Pre-Hospital and Emergency, SG Giuseppe Moscati Hospital, 74100 Taranto, Italy; mario.balzanelli@gmail.com (M.G.B.); distratispietro@gmail.com (P.D.); rita-lazzaro@libero.it (R.L.); 2 Department of Interdisciplinary Medicine, University of Bari “Aldo Moro”, 70124 Bari, Italy; giannadipalma@tiscali.it (G.D.); luigi.vimercati@uniba.it (L.V.); ad.inchingolo@libero.it (A.D.I.); m.e_maggiore@yahoo.it (M.E.M.); dr.antonio.mancini@gmail.com (A.M.); angeloinchingolo@gmail.com (A.M.I.); drkieukaren@gmail.com (K.C.D.N.); francesco.inchingolo@uniba.it (F.I.); 3 Director Multidisciplinary Research Center, Lincoln University, Oakland, CA 94612, USA; aityan@lincolnuca.edu; 4 Department of Emergency and Organ Transplantation, University of Bari “Aldo Moro”, 70124 Bari, Italy; ritalaforgia@hotmail.it (R.L.); angela.pezzolla@uniba.it (A.P.); 5 HOLOS Medical and Research Center, 00071 Pomezia, Italy; dietomoh@gmail.com; 6 Foundation of Physics Research Center, 87053 Celico, Italy; 7 “Phan Chau Trinh” University of Medicine and Nam-Khoa Biotek, Ho Chi Minh 50000, Vietnam; van.pham@pctu.edu.vn; 8 SET-118, Department of Pre-Hospital and Emergency, BAT, 76121 Barletta, Italy; donato.iacobone@aslbat.it; 9 Post Acute COVID-19 Department, “Umberto I” Hospital Unit-PTA, 74017 Mottola, Italy; annalisa.castrignano@asl.taranto.it; 10 Department of Innovative Technologies in Medicine and Dentistry, University of Chieti-Pescara, 66100 Chieti, Italy; ascarano@unich.it; 11 Department of Biomedical Science and Human Oncology, University of Bari, 70124 Bari, Italy; silvio.tafuri@uniba.it; 12 University Policlinic Hospital of Bari, 70124 Bari, Italy; giovanni.migliore@policlinico.ba.it; 13 American Stem Cells Hospital, Ho Chi Minh 70000, Vietnam; 14 Department of Histology-Embryology and Genetic, “Pham Ngoc Thach”—University of Medicine, Ho Chi Minh 70000, Vietnam; trancongtoai@pnt.edu.vn

**Keywords:** COVID-19, Sars-CoV-2, CD34+ hematopoietic stem cell (HSCs), thrombosis, thrombophilia, angiotensin-converting enzyme 2 (ACE2)

## Abstract

To date, several cases of thrombosis have been confirmed to be related to Sars-CoV-2 infection. Multiple attempts detected the prolonged occurrence of Sars-CoV-2 viral RNA (long COVID) in whole blood suggesting that virus byproducts may remain within cells and tissues well over the disease has finished. Patients may develop severe thrombocytopenia, acute anemia of inflammation and, systemic thrombosis with the fatal course of disease, which is suggestive of further interferences of Sars-CoV-2 on hematopoietic stem cells (HSCs) within the differentiation process towards erythroid and megakaryocytic cells. Therefore, we speculated whether Sars-CoV-2 propagates in or compartmentalizes with hematopoietic progenitor, erythroid, and megakaryocytic cells as the main cause of thrombotic events in either COVID-19 patients or vaccinated individuals. Results: The Sars-CoV-2 RNA replication, protein translation and infectious particle formation as the spike proteins in hematopoietic cell lines take place via the angiotensin-converting enzyme 2 (ACE2) entry pathway within primary CD34^+^ HSCs inducing, ex vivo, the formation of defected erythroid and megakaryocytic cells that eventually become targets of humoral and adaptive immune cells. Conclusions: Viral particles from affected CD34^+^ HSCs or the cellular component of RBC units and eventually platelets, present the greatest risk for sever thrombosis-transmitted Sars-CoV-2 infections.

## 1. The CD-34+ Hematopoietic Stem Cells and the Risk of Thrombocytopenia and Thrombotic Events in COVID-19 Infection, the Hypotheses of the Disturbances in the Myeloid Trait

Sars-CoV-2 virus, a member of the Coronavirus family, was recently isolated in 2019 in the city of Wuhan in Hubei province, People’s Republic of China, as the etiologic agent of coronavirus disease 2019 (COVID-19). The Sars-CoV-2 virus caused several large outbreaks in Asia before it spread through Europe, the Americas and India [1,2]. The majority of infected people remain asymptomatic or develop mild symptoms such as fever, cough, conjunctivitis, myalgia, fatigue and, minor neuropathies [3]. However, more severe complications such as Guillain-Barré syndrome in adults and elderly have been associated with Sars-CoV-2 infection and led to the declaration of COVID-19 as a *Public Health Emergency of International Concern* by the World Health Organization (WHO) in 2020 [4,5,6].

Data from different studies confirmed a drastic drop in platelet count (thrombocytopenia), microcytic anemia, low iron, and a fatal outcome for a patient with sickle cell anemia. These observations raised the question whether Sars-CoV-2 has the capability to infect hematopoietic stem cells interfering in the myeloid differentiation process and particularly those cells belonging to the erythroid and megakaryocytic lineage [7].

**Figure 1:**
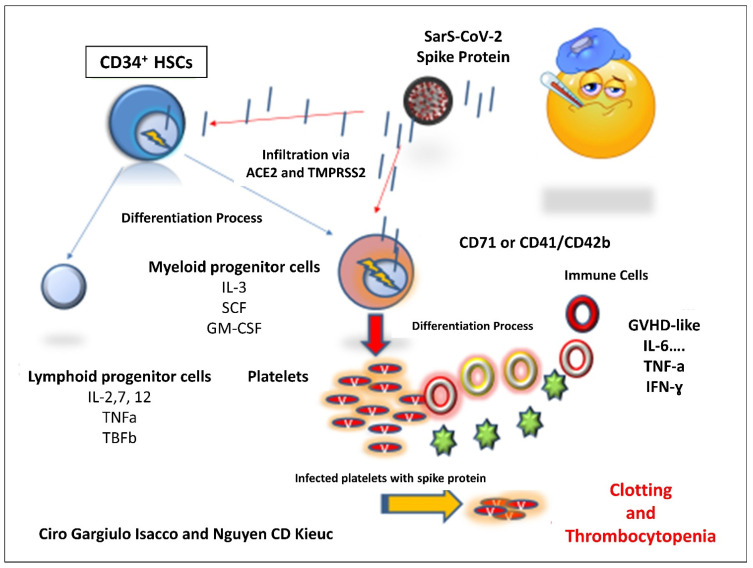
The etiopathogenesis of thrombotic event caused by the S proteins released by the Sars-CoV-2 virus. The current hypothesis is substantially based on three momenta: (i) The subversive activity perpetrated by the virus at the very beginning of the initial differentiation phase of CD34+ HSCs towards the myeloid lineage. (ii) The presence of SNPs at various level of the immune system’s both inflammatory and modulatory response. (iii) SNPs present of gene controlling the coagulation system (MTHFR and Leiden).

During hematopoiesis, multipotent human HSCs and progenitor cells, characterized by the expression of a cluster of differentiation 34 (CD34), are able to differentiate into several lineages, including common myeloid cells and lymphoid progenitors (Figure 1). Myeloid progenitor cells differentiate to megakaryocyte/erythroid lineage or granulocyte/macrophage progenitor phenotypes. 

The entire process of differentiation to erythrocytes, megakaryocytes, and megakaryocyte-derived platelets is mainly driven by erythropoietin (EPO) and thrombopoietin (TPO) [8,9]. During the differentiation process, the HSCs lose the distinctive CD34+ marker; instead, the erythroid and megakaryocytic differentiation is characterized by the appearance of CD71/Glycophorin A (GLYA) or CD41/CD42b (Figure 1) on erythrocytes and megakaryocytes/platelets, respectively [10,11,12].

Sars-CoV-2 was reported to infect leukocytes—mainly monocytes and macrophages—and B and T cells subtypes—mainly CD4, CD4 naïve, CD8 naïve, T suppressor (CD8-CD57) and T regs (CD4+CD25+^high^) [13]. However, so far, little is known about the infection of CD34^+^ HSCs or platelets phenotype by Sars-CoV-2.

Nevertheless, the mechanism of Sars-CoV-2 infection is well understood and could presumably work for any types of cells. The binding between the viral surface spike glycoprotein (S) and target cell surface takes place via the angiotensin-converting enzyme-2 (ACE2), followed by the cleavage of S by the transmembrane protease serine 2 (TMPRSS2). Several research teams observed that this mechanism via ACE2 could also be observed in intestinal epithelial cells, hepatocytes, and neurons, which explains the multiplicity of symptoms that characterize COVID-19 disease [13,14]. Since recently, an aberrant increase in erythroid progenitors together with an aberrant decrease in platelets circulation has been showed either in critical hospitalized cases or after receiving the vaccine shot. Indeed, these observations together with hypoxia, hypocapnia, alkalosis, iron anemia, and coagulopathies were seen highly correlated with an alarming grade of death risk [15,16,17]. Of note, progenitors of the erythroid and myeloid lineage appear to be the only cell types expressing both ACE2 and TMPRSS2 among the cells present in bone marrow. Medical reports from COVID-19 patients showed an increased mean platelet volume (MPV) and platelet hyperactivity, which were occasionally associated with a decrease in the overall platelet count. In our experience, detectable Sars-CoV-2 RNA in the blood stream was associated with platelet hyperactivity in critically ill patients.

Zhang and colleagues clearly demonstrated that platelets are able to express ACE2 and TMPRSS2; they also described the mechanism by which Sars-CoV-2 spike proteins induce platelet hyper-activation and super-aggregation via PAC-1 binding, CD62P expression, α granule secretion and dense granule release, thereby enhancing thrombosis formation [18].

**Figure 2:**
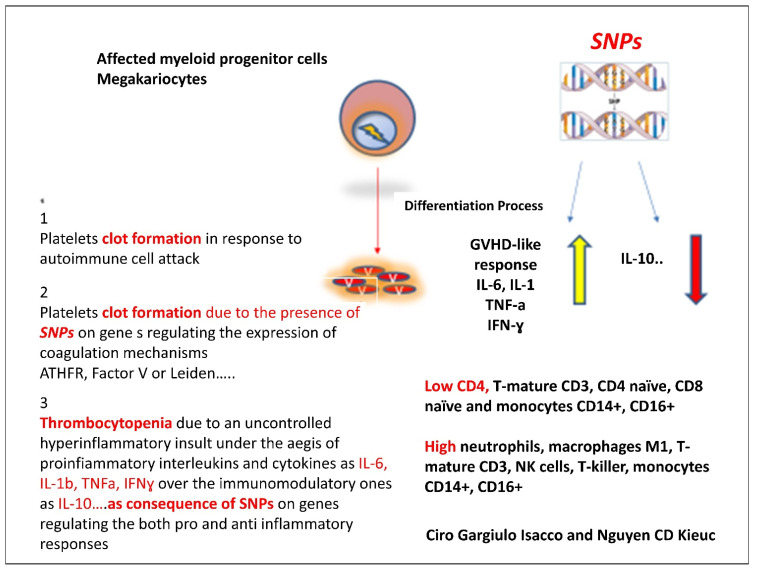
The capacity of the Sars-CoV-2 of interfering the differentiation process from CD34+ to megakaryocytes tend to generate a lineage of affected platelets that become target of autoimmune response via macrophages-M1, neutrophils, T-killers that in turn increase the production of pro-inflammatory cytokines and interleukins IL-6, IL-1β, TNFα and INFγ. The impossibility of reversing the process is probably due to two main factors; first, the presence of SNPs on genes regulate the expression of coagulative factors such as MTHFR and Leiden factor; second, the SNPs present on genes regulate the inflammatory responses (IL-6, IL-1β, TNFα and INFγ) and genes conversely regulate the immunomodulatory responses (IL-10, TGF-β).

Although they provided clear evidence suggesting the role of the MAPK pathway, downstream of ACE2, enhancing the magnitude of Sars-CoV-2 on platelet activation, in releasing coagulation factors, inflammatory factors, and in the formation of leukocyte–platelet aggregates, we assume otherwise that this is not comprehensive. In fact, the coagulation process was well described by Di Castelnuovo and colleagues referring to the hypercoagulable condition as a possible pathogenic mechanism contributing to disease progression and lethality in COVID-19 hospitalized patients [18]. The insurgence of rare thrombosis events either in COVID-19 affected patients or in post-vaccinated individuals needs further consideration. In our opinion, four factors should be considered: (i) the formation of the “abnormal” platelet progenies from Sars-CoV-2-infected myeloid megakaryocytes which become targets of host immunity (T cells, Th1, NK cells, macrophage M-1); (ii) the presence of single nucleotide polymorphisms (SNPs) on genes regulating the coagulation cascade such as MTHFR and Leiden-factor V; (iii) SNPs at the level of genes regulating the expression of both pro-inflammatory responses (IL-6, TNFα, IFNγ) and immune-modulatory responses (IL-10); (iv) fast platelets turnover with an average lifespan of 8–10 days (Figure 1 and Figure 2).

Several viruses showed this capability of interfering during the myeloid differentiation process to platelets or erythrocytes mainly in consequence of their fast turnover and due to their short half-life. The example may come from idiopathic thrombocytopenic purpura (acute or chronic) that may follow a viral illness, such as chickenpox or Zika virus. In Zika infection, authors observed that cell contagion started from the granulocyte/macrophage lineage, by which patients developed severe thrombocytopenia and microcytic anemia, stressing that the death risk may have followed in complications due to the interference of Zika virus with erythroid and megakaryocytic cells. According to the authors, the hematopoiesis phase is a crucial moment for the Zika virus to execute its opportunistic attack. This is the most critical phase as multipotent human hematopoietic stem and progenitor cells (HSPCs-CD34) start differentiating into several lineages, including common myeloid (CMP) and lymphoid (CLP) progenitors [19,20,21].

The contaminated platelet progenies become an easy and highly vulnerable target of the host immune cells. In addition, in response to an acute infectious phase, such as sepsis, platelets tend to be hyper-reactive promoting the formation of disseminated intravascular coagulation (DIC), which obstructs vessels leading to the formation of systemic ischemic blood clots and multiple organ failure [22,23]. In this scenario, platelets will be both the target and promoter of an uncontrolled pro-inflammatory cytokine storm and bind to neutrophils and release NETs, which in COVID-19 terminal patient promotes lung collapse and failure [22,23,24,25].

The presence of SNPs in cytokine and interleukin genes promotes a further aggravation of the symptoms. The variants not only could be related to the disease susceptibility and cytokine storm, but to COVID-19 complications as well. For instance, variants in *ACE2* and *TMPRSS2* have been associated to irreversible thrombotic risk condition, while variants in both IL-6 and IL-10, which regulate the level of inflammatory and immune modulatory response, may compromise the correct balance between the two phases leading to an uncontrolled irreversible inflammatory process. The low or total lack of the expression of IL-10 together with an overexpression of IL-6, TNFα and IFNγ as a consequence of polymorphisms attracts great attention especially in diseases following metabolic dysfunctions and in aging-related diseases, typical features of COVID-19-affected individuals. All this information open up a new discussion on COVID-19, especially if one considers the long COVID disease manifestation as it may exhibit different patterns with permanent organ damages and persistent post-COVID symptoms [26,27,28,29].

## 2. Validating the Premises

Albeit, it is yet scientifically to be demonstrated, there are raising concerns toward a possible association between SARS-CoV-2 and the potential breakdown of the hematopoietic stem cells differentiation process which causes thrombocytopenia, thrombolytic events and acute anemia. We proposed here in accordance with recent immunological advancements in COVID-19 research a new model of pathogenesis. This means that COVID-19 is a complex condition consequent of multiple local, systemic and genetic factors that worsen an individual’s correct response.

Several results and findings have validated this hypothesis. The analysis and measurement of serum CD34+ HSCs, affected megakaryocytes and platelets, the polymorphism on cytokines and interleukins, the over-expression of T-cells, neutrophils NET and M1 macrophages are all features that have been routinely found and assessed which eventually explain those death cases of devastating thrombosis as a consequence of Sars-CoV-2 and vaccine-released S proteins.

In addition, due to its close relation to Zika and Dengue virus present in the Asia Pacific region, the Americas and the Caribbean, the Sars-CoV-2 virus similarly showed its association with significant clinical manifestations such as neural damages and the Guillain-Barré Syndrome, a tropism that has been seen in the context of hematopoietic stem cells activity [30]. We also speculated that Sars-CoV-2-induced impairment of blood vessel integrity or development is mainly related to the high degree of inflammation due to the presence of specific polymorphisms (SNPs) on genes in charge of regulating inflammatory and immune-modulatory responses such as IL-6, IL-1a;b, TNF-α, IFN-γ and IL-10. However, further investigations are crucial to unravel the connection of Sars-CoV-2 pathogenicity and symptom manifestation towards prevention and therapy.

## Data Availability

All experimental data to support the findings of this study are available from the corresponding author upon request.
